# Detection of Anti-LipL32 Antibodies in Serum Samples from Horses with Chronic Intraocular Infection with *Leptospira* spp.

**DOI:** 10.3390/pathogens10101325

**Published:** 2021-10-14

**Authors:** Tobias Geiger, Hartmut Gerhards, Bettina Wollanke

**Affiliations:** 1 Clinic for Horses, University of Veterinary Medicine Hannover, 30559 Hanover, Germany; tbsgeiger@googlemail.com; 2 Equine Clinic, Clinical Department, Ludwig Maximilians University, 80539 Munich, Germany; gerhards@lmu.de

**Keywords:** equine recurrent uveitis, ERU, *Leptospira* spp., diagnostics, micro agglutination test, MAT, LipL32, SNAP Lepto, serum samples

## Abstract

Equine recurrent uveitis (ERU) is typically caused by chronic intraocular leptospiral infection in warm-blooded horses in central Europe. The most effective therapy for leptospiral-induced ERU is the surgical removal of diseased vitreous (vitrectomy). Since vitrectomy is a highly specialized and invasive surgery, the indication must be determined very carefully. In order to obtain evidence of intraocular leptospiral infection by laboratory diagnostics in questionable leptospiral ERU-cases, sampling of aqueous humor is required, because serum tests using microscopic agglutination test (MAT) are too unspecific. The SNAP Lepto is a cross-species rapid test for the detection of anti-Lipl32 antibodies that has a high sensitivity (0.97) and specificity (1.00) for the detection of anti-leptospiral antibodies using aqueous humor or vitreous samples, which is comparable to MAT. To evaluate sensitivity and specificity of SNAP Lepto using serum, serum samples from 90 horses with confirmed leptospiral ERU and from 103 ocularly healthy horses were tested by both MAT and SNAP Lepto. Sensitivity was similar for both tests (0.82 vs. 0.79), but specificity was lower for MAT (0.52 vs. 0.95). Sensitivity and specificity are therefore lower in serum samples compared to intraocular samples, however, the SNAP Lepto is far superior to MAT and suitable as a screening method using equine serum.

## 1. Introduction

In central Europe, equine recurrent uveitis (ERU) with its classic symptoms is typically caused by a chronic intraocular leptospiral infection [1,2,3,4,5,6,7,8,9,10,11]. In the following, the term "ERU" will therefore be used for leptospiral induced recurrent uveitis in warm-blooded horses with painful episodes, and which has been demonstrated to be a chronic intraocular infection. Recently, it has been demonstrated that infectious leptospiral uveitis is accompanied by biofilm formation [12,13].

ERU is a late sequela of systemic leptospirosis and becomes clinically apparent from about 6 months to several years after systemic leptospirosis [6,14,15,16,17,18,19,20,21,22,23,24,25,26,27]. The most effective therapy for ERU is vitrectomy of the diseased eyes [11,28,29,30,31,32,33,34,35,36,37,38,39,40]. Vitrectomy is used to eliminate the intraocular leptospiral infection so that no further ERU attacks occur in more than 95% of operated eyes [11,33,35]. If the surgical course is uncomplicated and if vitrectomy is performed before irreversible damage to the lens and/or retina has occurred due to ERU, vision can be preserved [11,33]. The most frequently detected serovar in ERU is Grippotyphosa (Supplementary 1, Figures S1 and S2).

In most cases, both the history and the ophthalmologic findings are conclusive in an ERU, so that the indication for vitrectomy can be reliably made [11,33,35]. In other cases, where the history is suggestive of ERU but the ophthalmologic findings are questionable, the indication for vitrectomy cannot be reliably established by ophthalmologic examination. Examination of serum by micro agglutination test (MAT) unfortunately does not allow a reliable statement about a local leptospiral infection in the eye, because too many healthy horses in Europe [5,6,10,41,42,43,44,45,46,47,48] as well as in other parts of the world [24,27,49,50,51,52,53,54,55,56,57,58,59,60,61] have agglutinating antibodies in the serum.

Therefore, an antibody titer in a serum sample determined with MAT has no significance for the diagnosis of ERU in an individual horse [1,3,4,5,6,10,62,63,64,65]. Consequently, serum tests using MAT do not allow a careful decision on the indication for surgery. However, since vitrectomy is a highly specialized and demanding ophthalmosurgical invention and complications can lead to blindness of the eye and even can make enucleation necessary. Thus, the correct indication is crucial.

For this reason, aqueous humor testing is indicated preoperatively in questionable ERU cases [5,6,11,65,66,67,68,69]. If either anti-*Leptospira* antibodies are detectable in the aqueous humor and/or the LipL32 gene of pathogenic *Leptospira* spp. can be detected by PCR, there is an indication for irrigation of the vitreous cavity. To avoid the relative invasive aqueous humor sampling for preoperative laboratory tests, laboratory methods for testing serum samples, which are less complicated to obtain than aqueous humor samples, need to be improved.

The SNAP Lepto, a rapid ELISA test has been commercially available for a few years. It is not species-specific and detects antibodies of different immunoglobulin classes directed against LipL32. LipL32 is a lipoprotein which is expressed at high levels by pathogenic *Leptospira* spp. [70]. Anti-LipL32 antibodies have been shown to be highly specific for the detection of infection with pathogenic *Leptospira* spp. [70]. In addition to its strong immunogenicity, LipL32 is also present in all pathogenic *Leptospira* spp. [71].

This quick ELISA test has proven to be very reliable for the examination of intraocular samples (aqueous humor and vitreous material) [69]. When testing intraocular specimens, the sensitivity and specificity of this rapid test are equivalent to those of the MAT [68], with a kappa value of 0.735 for MAT and SNAP tests [69]. The purpose of the present study was to examine the results of the SNAP Lepto test when serum samples were used and to compare the results of the MAT and SNAP Lepto test. The hypothesis was that using serum samples, the results of the SNAP Lepto test would be similar to those of the MAT and, thus, would not provide any additional information regarding local intraocular leptospiral infection in an individual horse.

## 2. Materials and Methods

### 2.1. Preliminary Examination and Classification of Equine Patients

Samples from 207 horses were used for the study. One hundred and three of the 207 horses in which neither the history nor the ophthalmologic examination revealed hints for ERU were considered to be ocularly healthy. Ninety horses had been diagnosed with ERU, and 14 horses with a leopard coat pattern (Appaloosas and Knabstruppers) had a history of chronic insidious uveitis without previous painful episodes of inflammation.

In the ocularly healthy horses, no intraocular samples but only serum samples were examined.

In the horses suffering from ERU, aqueous humor had either been collected during preoperative diagnostics in order to confirm the indication for vitrectomy on the basis of laboratory tests, or vitrectomy had been performed on the basis of the history and ophthalmologic findings without first collecting aqueous humor. If vitrectomy has been performed without previous aqueous humor testing, vitreous material obtained during therapeutically indicated surgery was used for laboratory diagnosis. The vitreous samples were collected at the beginning of each operation to avoid dilution of the samples by the irrigation fluid as far as possible. Horses were assigned to the ERU group if either in the aqueous humor samples or in the vitreous samples an antibody titer against a leptospiral serovar of 1:100 or higher was detectable by MAT, if the SNAP Lepto test was positive, or if the LipL32 gene was detectable in the intraocular samples by real-time PCR (target LipL32 gene, positive if Ct ≤ 40) (Supplementary 2).

In each case, aqueous humor was collected from the leopard coat pattern horses to determine a possible indication for vitrectomy. Although leopard coat pattern uveitis is not *Leptospira*-induced in most cases [72], meaning the eyes usually do not benefit from vitrectomy, intraocular leptospiral infection may be present in these horses in individual cases. Therefore, it was indicated to take aqueous fluid

SNAP Lepto was performed on-site as previously described [68]. MAT and PCR were performed at an external laboratory (IVD GmbH, Society for Innovative Veterinary Diagnostics, 30926 Seelze-Letter, German accreditation authority DAkkS, DIN EN ISO/IEC 17025, D-PL-18303-02-00; Reg.-Nr.: SAL-BY-L20-04-03). For MAT, the serovars Australis (Serogroup Australis), Bratislava (Serogroup Australis), Autumnalis, Canicola, Grippotyphosa, Copenhageni (Serogroup Icterohaemorrhagiae), Icterohaemorrhagiae (Serogroup Icterohaemorrhagiae), Pomona (Serogroup Pomona), Altodouro (Serogroup Pomona), Hardjo (Serogroup Sejroe), Saxkoebing (Serogroup Sejroe) and Tarassovi were used. A MAT titer of ≥ 1:100 was considered “positive” [73,74,75]. (Supplementary 3, Figures S3 and S4). 

### 2.2. Collection of the Serum Samples

Serum samples were available from all 207 horses. The serum either came from blood samples whose collection had been indicated preoperatively for other reasons, or blood was collected when the venous catheter was inserted for anesthesia (blood always drips off when the catheter is advanced so that the correct position can be checked). Thus, no vein was punctured specifically for obtaining the serum samples. The serum was allowed to stand for about one hour, then centrifuged (5 min, 2500× *g*) and decanted. Approximately 1 mL of serum from each horse was used for the present study. Few samples were frozen at −28 °C for a few days, most samples were directly examined and sent to the external reference laboratory immediately afterwards.

### 2.3. Examination of Serum Samples

#### 2.3.1. Preliminary Examination for the Use of SNAP Lepto

Preliminarily, 141 serum samples obtained for previous other studies (from horses not included in this study) and stored at −30 °C were examined with the goal to establish a baseline for agreement between MAT and SNAP Lepto test. For these samples, no consideration was given to the history of the horses. The serum samples were all from equine surgical patients and were tested for the presence of anti-LipL32 antibodies using the SNAP Lepto test (IDEXX company, Ludwigsburg, Germany) which was performed as previously described [68]. Any blue coloration of the sample spot, even a very slight one, was considered “positive”. The same serum samples were then sent to the IVD laboratory (see above) for MAT and tested for antibodies against the mentioned serovars. The result of the MAT was considered "positive" if the antibody titer against one serovar or more serovars was at least 1:100.

#### 2.3.2. Examination of the 207 Horse Sera of this Study

After obtaining serum, the SNAP Lepto test was first performed on-site. Subsequently, the remaining serum was shipped to the IVD-laboratory. The SNAP Lepto test and MAT were performed exactly as for the other serum samples.

### 2.4. Statistical Analysis

The data from the preliminary examination (141 serum samples) and the results from the 207 horse sera of this study were coded in Microsoft Excel 2011 and then analyzed in SPSS 25. Data collection took place between 2017 and 2019. Pearson’s chi-square test was used to determine dependencies and statistical correlations. The null hypothesis was defined as the independence of two variables of the four-field table. The significance level in this case was *p* = 0.05. The laboratory tests (MAT, SNAP Lepto and PCR) were evaluated for statistical agreement using the kappa value.

## 3. Results

### 3.1. Preliminary Examination: Use of MAT and SNAP Lepto for Equine Serum Samples

In 104 of the 141 serum samples (74%), a titer of 1:100 or higher was measured. In the sera in which no anti-Leptospira antibodies were detectable by MAT (titer < 1:100), no anti-LipL32 antibodies were detectable by SNAP Lepto either in most cases (32/37 sera, 86.5%) (Table 1). The agreement of both tests for a negative response was significant (Pearson’s chi-square test, *p* < 0.001). A MAT titer of ≥1:100 against one serovar or multiple serovars was present in 104 of the 141 sera (74%). In contrast, among the sera that reacted positively in MAT, antibodies directed against LipL32 could be detected by SNAP Lepto in only 59 of these 104 sera (56.7%). In 45 of the 104 sera positive in MAT (43.3%), no antibodies directed against LipL32 were detectable by SNAP Lepto. Thus, the agreement regarding a positive result in MAT and SNAP Lepto test was low (kappa value 0.34).

### 3.2. Examination of the 207 Horse Sera of This Study

Comparing the results of MAT and SNAP Lepto, it can be seen that while MAT is positive in over 80% of horses with ERU, it is also positive in almost half of the ocularly healthy horses (Table 2). The SNAP Lepto, on the other hand, is negative in over 90% of ocularly healthy horses and positive in nearly 80% of horses with ERU. The difference between the ocularly healthy horses and horses suffering from ERU when examined by the SNAP Lepto test is significant (Pearson’s chi-square test, *p* < 0.001). All 14 sera from horses with leopard coat patterns had a negative SNAP Lepto result when the serum samples were tested.

For the calculation of sensitivity, specificity, positive and negative predictive values, the ocular healthy horses and the horses with leopard coat pattern were combined in one group (“no ERU”) (Supplementary 4, Tables S1–S3). The sensitivity of serum tests with respect to detecting intraocular leptospiral infection was similar for MAT and SNAP Lepto (0.82 and 0.79, respectively). However, the specificity when using the SNAP Lepto was significantly higher at 0.95 compared to the MAT, which had a specificity of only 0.52. A similar difference was found for the positive predictive value, which was 0.92 for SNAP Lepto and 0.57 for MAT. The difference was less for the negative predictive value (MAT 0.79; SNAP Lepto 0.85). Statistical agreement for SNAP Lepto using serum and ERU was high (kappa value 0.76).

**Figure 1:**
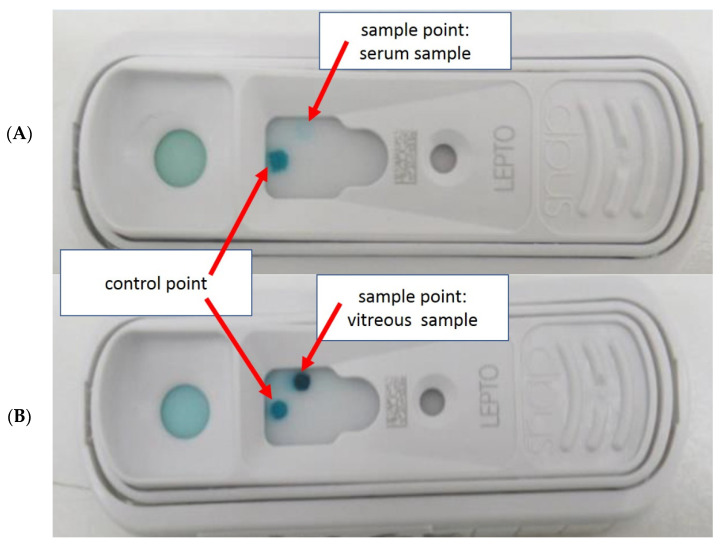
Faint blue sample point (**A**) of the serum sample and dark blue sample point (**B**) when examining the corresponding vitreous sample from the same horse.

Noticeably, the blue coloration of the sample spot of SNAP Lepto was usually less intense in the serum samples than in the corresponding intraocular samples (Figure 1).

## 4. Discussion

Once again, as expected, MAT with serum samples was found to be too unspecific for the diagnosis of intraocular leptospiral infection in this study. Eighty-five percent of sera from horses affected with ERU reacted with a titer ≥1:100 in MAT, but MAT titers ≥1:100 were also detectable in approximately half of the samples in the sera from the ocularly healthy horses. Thus, the specificity of MAT with equine serum samples is too low (0.52) to reliably predict the presence of intraocular leptospiral infection in individual horses.

Serum tests for anti-*Leptospira* antibodies have been frequently performed in ocular healthy horses and horses suffering from ERU [5,41,48,61,64]. Most of these studies used MAT, which has long been considered the gold standard in serum diagnostics [70,76,77], is cited in almost all studies as the reference method for the humoral immune response to leptospiral infection, and is still listed by WHO as the only reference method for screening tests [75,78]. The MAT is a challenging test, requiring some professional experience to perform and numerous serovars to keep available [70,74,75,76,77].

The fact that the examination of numerous horse sera in some studies has shown a difference between the MAT results of the group of ocularly healthy horses compared with those affected with ERU [21,41,49,79,80] does not change the fact that the examination of serum samples by MAT is not an appropriate method to diagnose *Leptospira*-induced uveitis in an individual horse. MAT lacks specificity because the background level of exposure in the equine population is too high in most studies [4,6,10,48,62,64].

Regardless of whether equine serologic studies were purely epidemiologic or focused on an association between *Leptospira* spp. and ERU, the results of the studies available in the literature vary considerably. The percentage of horses that had anti-*Leptospira* antibodies in serum using MAT was 1.5% in one study [44], but up to over 80% in other studies [6,53,57]. In numerous other studies, the frequency of seropositive horses is in between [43,46,48,58]. Using a specific ELISA test for detecting anti-*Leptospira* antibodies, even in up to 98% positive-reacting sera, has been detected in horses affected with ERU [63].

The different results in publications on serological testing of horse sera for anti-*Leptospira* antibodies can be explained, on one hand, by the fact that horses were exposed to different infection burdens, having lived in different geographic regions, countries, climatic zones, and environmental conditions and having been tested in different years. However, the MAT titer, which is considered "positive", also plays an essential role, being ≥1:50 [53,58], ≥1:100 [6,9,10,42,43,49,51,54,57,62,81,82,83], ≥1:400 [80,84,85,86] and even ≥1:800 [87] in different studies. Furthermore, different serovars were also used for MAT in different surveys. Although reference laboratories usually have serovars that are relevant to human medicine for the region in question, other species may harbor other serovars that were not tested for. The considerably varying number of serovars used in each case may also have influenced the different results. For example, the MAT included five serovars [58] in one study and 28 serovars [51] in another one. In the present study, the cut-off titer for MAT (1:100) was consistent with most recent studies and the current recommendations [88]. The number of serovars used is average and includes the serogroups relevant in Germany and neighboring countries.

In contrast to MAT, the ELISA rapid test (SNAP Lepto) provided an unexpected result in the present study. Anti-LipL32 antibodies were detected in the serum of 71 of the 90 (79%) horses affected with ERU, whereas in only 6% (6/103) of serum samples from horses with clinically healthy eyes anti-LipL32 antibodies were detected. Thus, the SNAP Lepto was particularly useful in detecting those horses that did not have a chronic leptospiral infection in the eye but had some history of recurrent eye disease. The SNAP Lepto is much more specific (specificity 0.95) than the MAT (specificity 0.52). Thus, the hypothesis that the use of the SNAP Lepto does not offer any advantage over the MAT to diagnose chronic intraocular leptospiral infection from serum samples was not confirmed.

The number of sera from horses affected with ERU examined in this study is, on average, similar to the numbers of other studies [10,21,64,79,89]. However, an expansion of the sample contingent would be beneficial for further substantiation of the present results, but is not achievable within an acceptable time frame in equine medicine.

The 6% of horses with clinically healthy eyes in which anti-LipL32 antibodies were detectable in the present study correspond approximately to the incidence of horses developing ERU in Germany [90]. In other studies, positive PCR results (detection of the LipL32 gene) had been described in 5% [83,91], 3% [92], and 0.4% [68] of intraocular specimens from clinically healthy eyes. These PCR-positive intraocular specimens suggest asymptomatic leptospiral infection of the eye. It is not possible to determine whether the horses with clinically healthy eyes from which the SNAP Lepto positive serum samples were obtained would have developed clinically recognizable symptoms in terms of ERU over the following months or years. Thus, the sera from ocularly healthy horses in this study that reacted "false-positive" in SNAP Lepto could have come from horses that had an intraocular leptospiral infection that had not (yet) resulted in clinical signs. The examination of intraocular samples from the clinically healthy horses was not possible in the present study for ethical reasons.

In few previous studies with serum samples from horses, different ELISA tests had already been used [55,63,65,93]. In horses, the detection of serovar-specific IgA antibodies using an in-house ELISA assay had been shown to be highly sensitive and highly specific for the diagnosis of intraocular leptospiral infection when intraocular samples were evaluated [63,65]. However, when serum samples are examined, IgA antibodies are also very often detectable in healthy horses [63,69]. Only in a species- and immunoglobulin-specific in-house ELISA test it was found that the simultaneous detection of antibodies of different immunoglobulin classes (IgM + IgA + IgG) was quite predominantly possible in horses suffering from ERU and hardly in horses with ophthalmologically healthy eyes [63]. However, the number of horses in the study was relatively small, so this relationship would need further investigation.

The humoral response to leptospiral infections has been thoroughly investigated, particularly in human medicine. In addition to agglutinating antibodies, antibodies against various dominant immunoreactive protein antigens are also produced in leptospiral infections, e.g., against the outer membrane lipoprotein LipL32 or the heat shock proteins GroEL and DnaK [70,94]. Although the detection of antibodies directed against LipL32 does not provide any information about the serovar causing the infection, this is irrelevant for the therapy. Antibodies against LipL32 are also detectable when the infection is caused by a serovar that is not available for MAT [76].

Different dynamics of agglutinating and other antibodies have been repeatedly described for humans and animals [70,74,94,95,96,97,98]. Especially in cases where the MAT is negative against clinical expectation, supplementary serological tests are of importance [75,76,77]. Various ELISA tests, among others, have been described as serological tests complementary to MAT [70,74,75,76,94,99]. Most commercially available ELISA tests detect immunoglobulin class M antibodies, which are critical for early diagnosis of acute infection and are often detectable before MAT becomes positive [75,76,78,98,100,101].

Not only in the very early stages of leptospirosis, but also in chronic leptospiral infections, MAT is less reliable than ELISA tests [76,78]. The current OIE manual indicates that MAT is an imperfect test in some chronic infections, having a sensitivity of less than 50% [88]. In horses, it has been shown that in 9.5% of horses in which *Leptospira* spp. were cultured in vitreous samples, the corresponding serum samples reacted negatively in MAT [6]. In hedgehogs and rodents in which PCR or culture had yielded a positive result when urine was examined, MAT was often negative when the corresponding serum samples were examined [102,103]. Here, it would be interesting to know whether anti-LipL32 antibodies would have been detectable.

In horses suffering from ERU, infections with different serovars from different serogroups could be detected by culture and MAT in previous studies when examining intraocular sample material [6,104,105], but this is therapeutically irrelevant. The advantage of the immunoglobulin-nonspecific SNAP Lepto used in the present study is that basically all antibodies directed against LipL32 (having at least 2 binding sites) are detectable, which increases the sensitivity compared with other immunoglobulin-specific or even serovar-specific ELISA tests, provided that the humoral immune response has led to the formation of anti-LipL32 antibodies.

The reason for the different results of MAT and SNAP Lepto in the present study may be that in horses, too, the agglutinating antibodies detectable by MAT have different dynamics in the course of leptospiral infections in horses than the antibodies directed against LipL32. The result of this study suggests that chronic local infection with *Leptospira* spp. in the eye persistently leads to the production of anti-LipL32 antibodies in most horses, which are almost exclusively detectable in the serum of horses affected with ERU and hardly detectable in horses with clinically healthy eyes. It is possible that the formation of antibodies directed against LipL32 declines more rapidly than agglutinating antibodies after a systemic *Leptospira* spp. infection that has not resulted in chronic local infection. The antibodies detectable in serum by MAT may also be due to intraocular leptospiral infection in horses suffering from ERU, but it is also possible that they represent a residual titer after previous leptospiral infection, completely independent of ERU. Thus, for MAT results it seems to be irrelevant whether the systemic leptospirosis has resulted in intraocular leptospiral infection or not.

The problems of diagnosing chronic intraocular infection with *Leptospira* spp. by serum testing in horses may be due not only to the immune privileged site in the eye and lack of immune responses, but also to biofilm formation of the bacteria inside the eye [13]. Uveitis caused by *Leptospira* spp. as a late consequence of systemic leptospirosis is also known in human medicine [106,107,108,109]. Here, too, diagnosis by serological tests is unreliable and the diagnosis of leptospiral uveitis can be challenging [110,111]. Thus, biofilm formation accompanying chronic leptospiral infection of the vitreous cavity might also be present in humans.

Detection of specific antibodies is also challenging in other biofilm-associated local infections, such as cystic fibrosis, and improvement of serological diagnostics is suggested [112,113,114]. It is interesting to note that the lower the immune response and the fewer antibodies are produced, the better the prognosis is often for people suffering from cystic fibrosis, as the inflammation in the tissues associated with the immune responses causes more significant damage than the infectious agents themselves [115,116,117]. This seems to be similar in ERU: higher intraocular anti-*Leptospira* antibody titers correlate with more severe intraocular inflammatory changes and, on the other hand, *Leptospira* spp. could be detected in culture even in specimens from ophthalmoscopically apparently healthy eyes in which no anti-*Leptospira* antibodies were detectable [5].

In chronic bacterial infections and especially in infections associated with biofilm formation, antibodies of the classes IgG and particularly IgA are of great diagnostic value [115,117,118,119]. IgA had also proven to be particularly sensitive and specific in the examination of intraocular samples from horses [65]. However, to the authors’ knowledge, IgA-specific ELISA tests for leptospiral serodiagnosis are currently hardly offered and are only available in the form of in-house tests with antigen preparations. In addition, they are time-consuming and expensive.

The results of the present study suggest that SNAP Lepto is a very good screening method for ERU when equine serum is examined. Particularly in questionable ERU cases, examination of serum by SNAP test may help to decide on the further course of action. If the SNAP Lepto with the serum gives a negative result, long transports of the horses to a clinic can be avoided. If the result of the SNAP Lepto is positive with serum, it is advisable to transport the horse to a clinic specialized in ophthalmology. There, on the basis of further ophthalmologic examinations (or, if necessary, an aqueous humor analysis), a decision can be made as to whether or not vitrectomy is indicated. Thus, the SNAP test can provide important information to veterinarians with little ophthalmologic experience that can help decide on further steps.

## 5. Conclusions

Results of the available samples have shown that serum testing using SNAP Lepto does not have the same high informative value as testing intraocular samples. Nevertheless, SNAP Lepto is significantly more informative than MAT when using serum samples. The results of this study indicate that the detection of anti-LipL32 antibodies in equine serum is very reliably indicative of chronic local (intraocular) infection with pathogenic *Leptospira* spp. (specificity 0.95) at an acceptable sensitivity (0.79). The positive predictive value for the detection of intraocular leptospiral infection was 0.92, and the negative predictive value was 0.85. Consequently, the SNAP test has proven to be a very good screening method for the practice to obtain evidence of ERU in otherwise questionable cases. Rapid tests detecting anti-LipL32 antibodies of different immunoglobulin classes could also prove valuable in other species.

## Figures and Tables

**Table 1:** Results of the analysis of 141 horse sera using MAT and SNAP Lepto.

		SNAP Lepto	
		Negative	Positive
MAT ^1^	negative	86.5%	13.5%
	(titer < 1:100)	(32/37)	(5/37)
	positive	43.3%	56.7%
	(titer ≥ 1:100)	(45/104)	(59/104)

^1^ Serovars: Australis (Serogroup Australis), Bratislava (Serogroup Australis), Autumnalis, Canicola, Grippotyphosa, Copenhageni (Serogroup Icterohaemorrhagiae), Icterohaemorrhagiae (Serogroup Icterohaemorrhagiae), Pomona (Serogroup Pomona), Altodouro (Serogroup Pomona), Hardjo (Serogroup Sejroe), Saxkoebing (Serogroup Sejroe) and Tarassovi.

**Table 2:** Results of serum sample testing using SNAP Lepto in ocular healthy horses, in horses suffering from ERU and in horses with leopard coat pattern uveitis (ERU: intraocular samples of these horses MAT positive and/or PCR positive).

	MAT Using Serum Samples		SNAP Lepto Using Serum Samples	
	Negative	Positive	Negative	Positive
no signs of ERU	51.5%	48.5%	92.2%	5.8%
(*n* = 103)	(53/103)	(50/103)	(97/103)	(6/103)
ERU	17.8%	82.2%	21.1%	78.9%
(*n* = 90)	(16/90)	(74/90)	(19/90)	(71/90)
uveitis in horses with leopard coat pattern	57%	43%	100%	0%
(*n* = 14)	(8/14)	(6/14)	(14/14)	(0/14)

## Supplementary Materials

The following are available online at https://www.mdpi.com/article/10.3390/pathogens10101325/s1, Supplementary 1: Short information about common serovars horses with ERU in Germany and neighboring countries, Figure S1: Comparing culture results of vitreous material (cultures from 187 eyes) and MAT results of serum samples, Figure S2: Culture results (*n* = 189) and MAT results with vitreous samples went together pretty well, Supplementary 2: Short explanation of uveitis in horses with leopard coat pattern, Supplementary 3: Laboratory results for the classification of patients, Figure S3: Preliminary examination, classification of patients: serovar distribution in intraocular samples using MAT, titers 1:100 or higher, Figure S4: Preliminary examination, results of MAT, PCR and SNAP Lepto in intraocular samples of ERU eyes, Supplementary 4: Results of serum examinations (Section 3.2.), Table S1: Results of MAT using equine Serum, Table S2: Results of SNAP Lepto using equine Serum, Table S3: Calculation of sensitivity, specificity, positive and negative predictive values (ppv and npv respectively) of MAT and SNAP Lepto using equine serum. References [120,121,122,123,124,125] are cited in the Supplementary Materials.
