# Supplementary material for: Detection of Anti-LipL32 Antibodies in Serum Samples from Horses with Chronic Intraocular Infection with Leptospira spp
Source: Pathogens. 2021 Oct 14;10(10):1325. doi: 10.3390/pathogens10101325 (PMC8537251; doi:10.3390/pathogens10101325)
Supplement: Supplementary file 1 [file pathogens-10-01325-s001.zip › pathogens-1408646-supplementary.pdf]

Supplementary 1. Short information about common serovars horses with ERU in Germany and neighboring countries.

In Germany and neighboring countries, serovar Grippotyphosa is detectable in about 80% of intraocular samples from ERU eyes. The field mouse is the assumed vector [120]. However, depending on the exposure of horses, intraocular infections with other serovars from different serogroups may also occur. Although MAT reactions with the serovars Icterohaemorrhagiae and Copenhageni are not infrequently detectable in serum samples, these serovars appear to play a lesser role, if any, in intraocular leptospiral infections [5,6,104,105] (Figures S1 and S2, presented on ELS 2018).

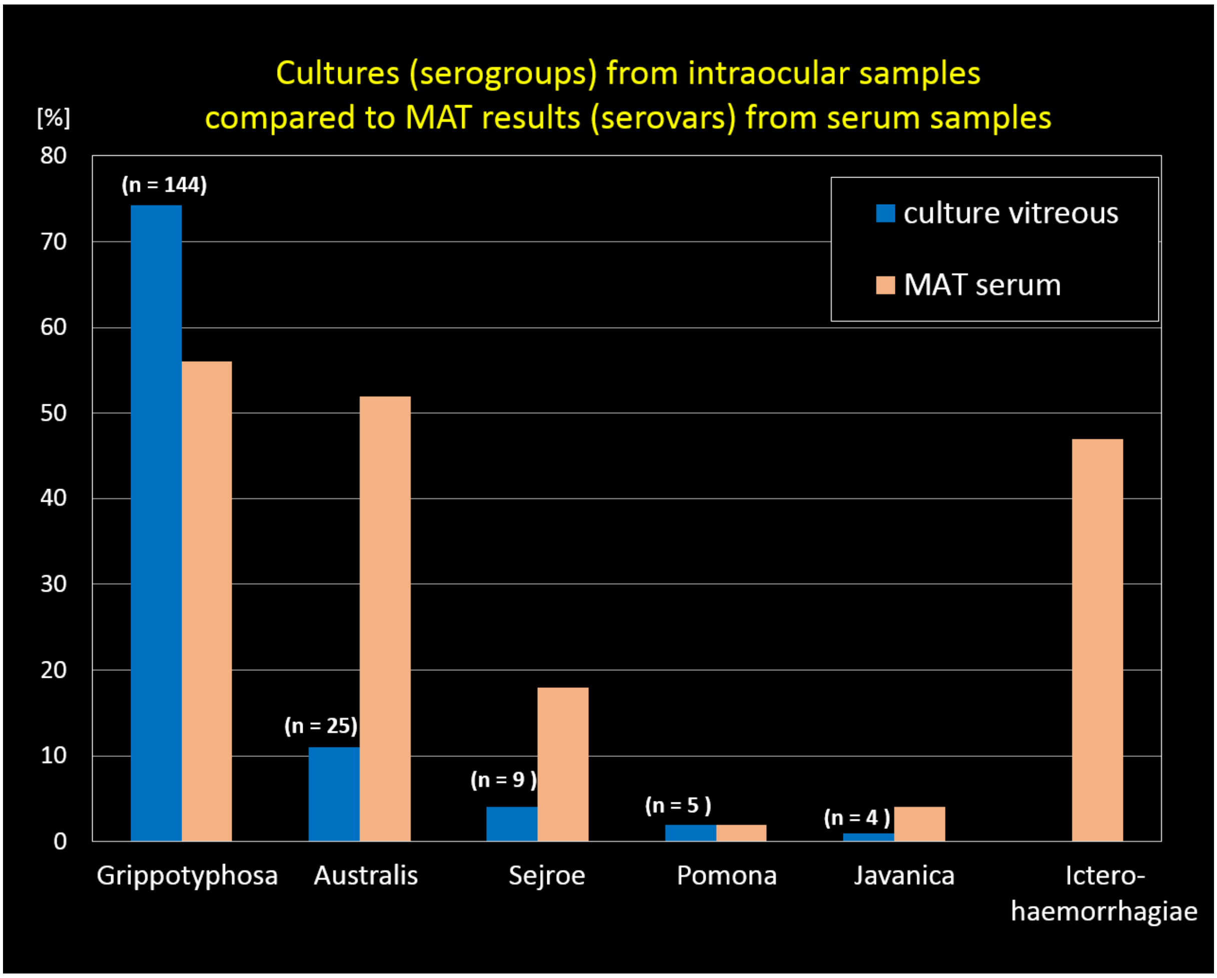

Figure S1. Comparing culture results of vitreous material (cultures from 187 eyes) and MAT results of serum samples: the results only partially go well together but differ especially looking at serogroups Australis, Sejroe and Icterohaemorrhagiae [105].

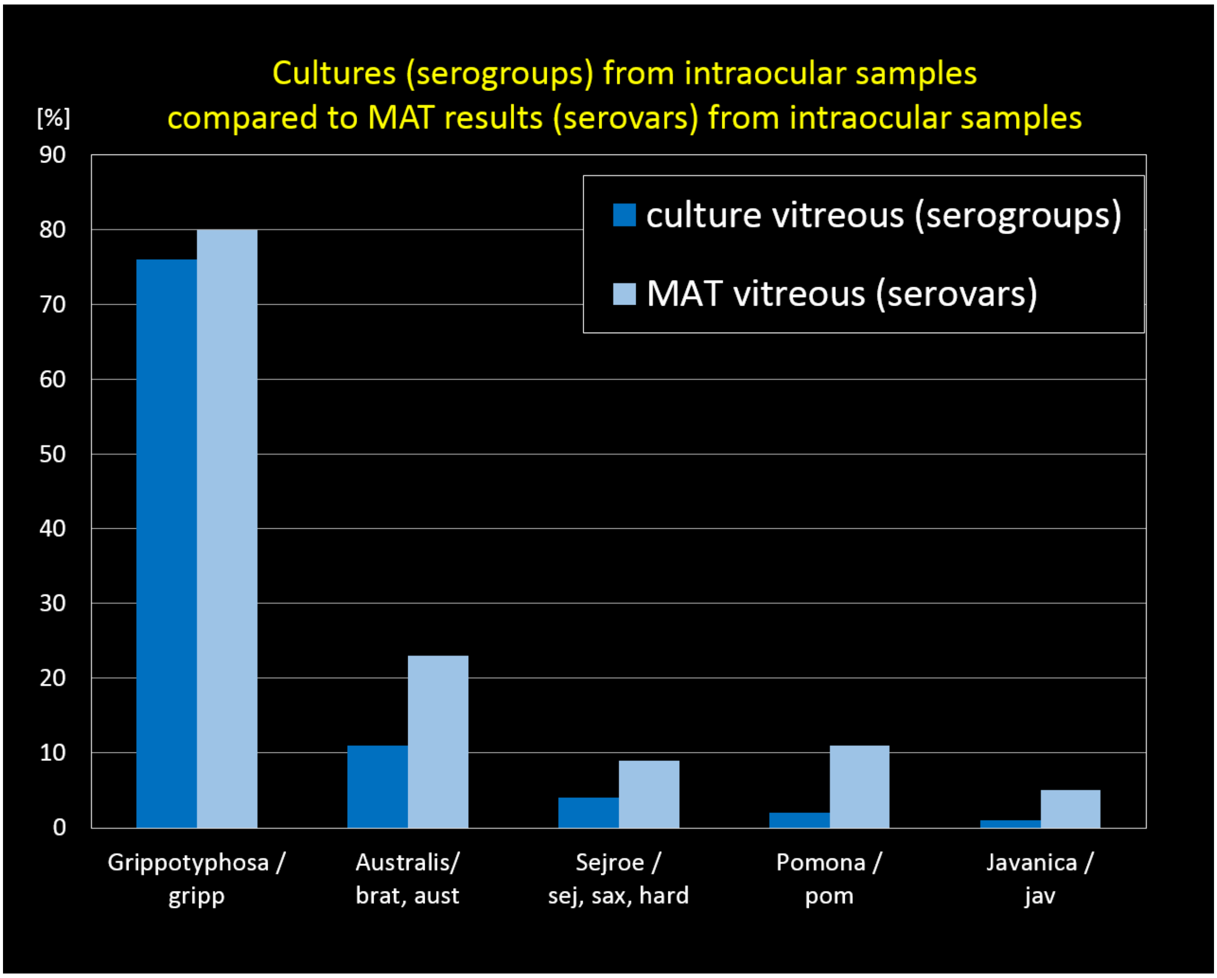

Figure S2. Culture results (n = 189) and MAT results with vitreous samples went together pretty well [105]. (Serovars: gripp = Grippotyphosa, brat = Bratislava, aust = Australis, sej = Sejroe, sax = Saxkoebing, pom = Pomona, jav = Javanica).

Supplementary 2. Short explanation of uveitis in horses with leopard coat pattern.

Horses with leopard coat pattern and especially Appaloosas typically show a persistent low-grade intraocular inflammation with a cumulative destructive effect. Appaloosas not only have an 8-fold increased risk of developing uveitis compared to other breeds, but most commonly both eyes are affected and the prognosis for preserving vision is poor. Uveitis is a particularly devastating disease in Appaloosas [72,121–125].

Supplementary 3. Laboratory results for the classification of patients.

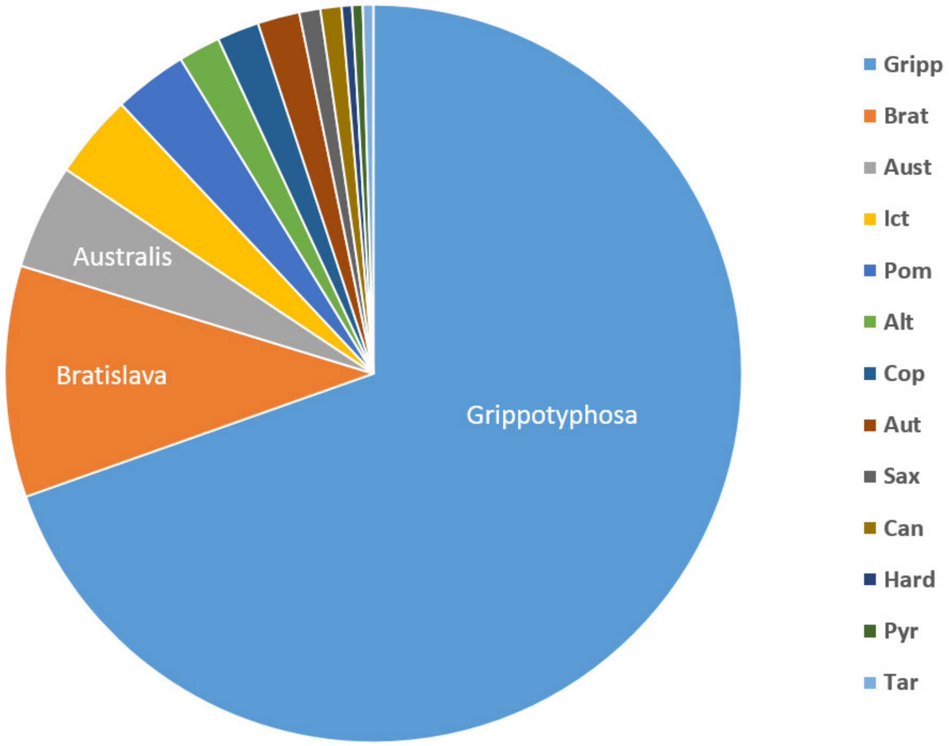

Figure S3. Preliminary examination, classification of patients: serovar distribution in intraocular samples using MAT, titers 1:100 or higher. Only the serovar with the highest titer is considered. (Gripp = Grippotyphosa, Brat = Bratislava, Aus = Australis, Ict = Icterohaemorrhagiae, Pom = Pomona, Alt = Altodouro, Cop = Copenhageni, Aut = Autumnalis, Sax = Saxkoebing, Can = Canicola, Hard = Hardjoe, Pyr = Pyrogenes, Tar = Tarassovi; serogroups see section 2.1.).

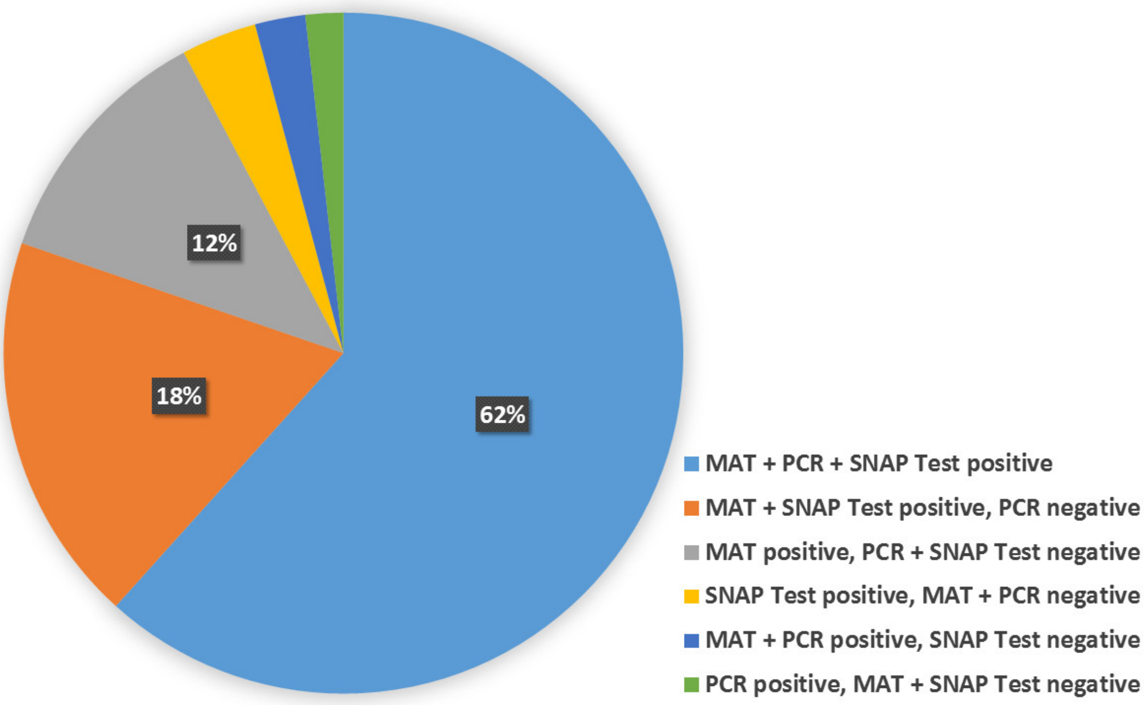

Figure S4. Preliminary examination, results of MAT, PCR and SNAP Lepto in intraocular samples of ERU eyes.

Supplementary 4. Results of serum examinations (section 3.2.).

Table S1. Results of MAT using equine Serum.

	ERU	No ERU [1]	∑
positive	74	50 + 6 = 56	130
negative	16	53 + 8 = 61	77
∑	90	117	207

[1] horses with healthy eyes + leopard coat pattern horses.

Table S2. Results of SNAP Lepto using equine Serum.

	ERU	No ERU[1]	∑
positive	71	6 + 0 = 6	77
negative	19	97 + 14 = 111	130
∑	90	117	207

[1] horses with healthy eyes + leopard coat pattern horses.

Table S3. Calculation of sensitivity, specificity, positive and negative predictive values (ppv and npv respectively) of MAT and SNAP Lepto using equine serum.

	Sensitivity	Specificity	ppv	npv
MAT	74/90 = 0.82	61/117 = 0.52	74/130 = 0.57	61/77 = 0.79
SNAP Lepto	71/90 = 0.79	111/117 = 0.95	71/77 = 0.92	111/130 = 0.85
